# The Association between Lifestyle Factors and COVID-19: Findings from Qatar Biobank

**DOI:** 10.3390/nu16071037

**Published:** 2024-04-03

**Authors:** Zoha Akbar, Hasna H. Kunhipurayil, Jessica Saliba, Jamil Ahmad, Layla Al-Mansoori, Hebah A. Al-Khatib, Asmaa A. Al Thani, Zumin Shi, Abdullah A. Shaito

**Affiliations:** 1 Department of Human Nutrition, College of Health Sciences, QU Health, Qatar University, Doha P.O. Box 2713, Qatar; 2 Biomedical Research Center, Qatar University, Doha P.O. Box 2713, Qatarh.alkhatib@qu.edu.qa (H.A.A.-K.);; 3 Department of Public Health, Faculty of Health Sciences, University of Balamand, Beirut P.O. Box 100, Lebanon; 4 Department of Biology, Faculty of Sciences, Lebanese University, Beirut P.O. Box 90656, Lebanon; 5 Hamad Medical Corporation, Doha P.O. Box 3050, Qatar; 6 Department of Biomedical Sciences, College of Health Sciences, QU Health, Qatar University, Doha P.O. Box 2713, Qatar; 7 Department of Basic Medical Sciences, College of Medicine, QU Health, Qatar University, Doha P.O. Box 2713, Qatar

**Keywords:** COVID-19, smoking, vitamin D, obesity, bariatric surgery, dietary patterns

## Abstract

Coronavirus Disease 2019 (COVID-19) manifestations range from mild to severe life-threatening symptoms, including death. COVID-19 susceptibility has been associated with various factors, but studies in Qatar are limited. The objective of this study was to investigate the correlation between COVID-19 susceptibility and various sociodemographic and lifestyle factors, including age, gender, body mass index, smoking status, education level, dietary patterns, supplement usage, physical activity, a history of bariatric surgery, diabetes, and hypertension. We utilized logistic regression to analyze these associations, using the data of 10,000 adult participants, aged from 18 to 79, from Qatar Biobank. In total, 10.5% (*n* = 1045) of the participants had COVID-19. Compared to non-smokers, current and ex-smokers had lower odds of having COVID-19 (odds ratio [OR] = 0.55; 95% CI: 0.44–0.68 and OR = 0.70; 95% CI: 0.57–0.86, respectively). Vitamin D supplement use was associated with an 18% reduction in the likelihood of contracting COVID-19 (OR = 0.82; 95% CI: 0.69–0.97). Obesity (BMI ≥ 30 kg/m^2^), a history of bariatric surgery, and higher adherence to the modern dietary pattern—characterized by the consumption of foods high in saturated fat and refined carbohydrates—were positively associated with COVID-19. Our findings indicate that adopting a healthy lifestyle may be helpful in the prevention of COVID-19 infection.

## 1. Introduction

Coronavirus Disease 2019 (COVID-19), caused by Severe Acute Respiratory Syndrome Coronavirus 2 (SARS-CoV-2), first emerged in late 2019 and was declared a global pandemic in March 2020. As of early November 2023, the World Health Organization (WHO) had recorded 771 million confirmed COVID-19 cases and over 6.97 million deaths worldwide [1]. COVID-19 exhibits a significant range of variations in its presentation among individuals affected by the virus. Some may remain asymptomatic throughout the course of infection, while others have symptoms that range from a mild upper respiratory tract infection to life-threatening pneumonia [2]. Those with a poor disease prognosis often suffer from Acute Respiratory Distress Syndrome (ARDS) or immune-mediated lung injury, which can be fatal [3]. COVID-19 disease severity may be attributed to immune dysregulation, leading to differences in immune response, stemming from but not limited to the risk factors of older age and comorbidities [4]. While anti-viral therapies and vaccines provide some protection against disease severity, emerging research suggests that lifestyle factors, such as dietary patterns and the use of dietary supplements, may play a role in mitigating disease severity through their anti-inflammatory and immune-modulating properties [5,6].

Numerous studies have demonstrated the association of sociodemographic and behavioral covariates with a higher risk of the acquisition of COVID-19 infection [7,8,9]. Age, gender, ethnicity, education, socioeconomic status, chronic diseases (e.g., hypertension, obesity, and diabetes), dietary patterns, and supplements have been associated with COVID-19 [10,11,12]. Among dietary supplements, vitamin D is the most extensively researched, where numerous studies have examined the association between vitamin D status and COVID-19 outcomes [6]. Associations of comorbid medical conditions with the acquisition of SARS-CoV-2 infection are less defined. However, diabetes and obesity are more robustly associated with SARS-CoV-2 infection [7,13,14,15,16].

The persistent and widespread effects of the COVID-19 pandemic, coupled with the emergence of new variants of the virus, emphasize the importance of further exploring the numerous factors that influence COVID-19 susceptibility and outcomes, especially in the Qatari population, which is plagued with a high burden of obesity and prevalence of bariatric surgery. Globally, Qatar holds the highest rate of bariatric surgery, with a prevalence as high as 12% [17].

Previous research has not explored the connections between lifestyle, sociodemographic factors, including a history of bariatric surgery, and susceptibility to COVID-19 in Qatar. This study aims to evaluate the relationship between COVID-19 and various lifestyle and demographic factors among adults, utilizing data from the Qatar Biobank (QBB). Additionally, we investigated whether a history of bariatric surgery is associated with COVID-19.

## 2. Materials and Methods

### 2.1. Design of Study and Sample Collection

This retrospective cross-sectional study analyzed the data of 10,000 participants in the QBB study. QBB is an ongoing population-based prospective cohort registry, which was established in 2012 by the Qatar Foundation and the Supreme Council of Health in collaboration with Hamad Medical Corporation (HMC). QBB aims to collect extensive biological, clinical, and lifestyle information on 60,000 men and women [18].

QBB participant recruitment takes place through the internet, social media, or family and friends. Upon recruitment, the participants are invited for a health examination at the QBB facility at HMC. The examination involves a self-administered questionnaire and a nurse interview. Sociodemographic data, lifestyle factors, and dietary habits are collected through questionnaires. In addition, interviews conducted by trained research nurses collect information on medical history, family history of disease, and use of medications. The participants undergo physical assessments, including anthropometric measurements such as weight and height *via* a Seca stadiometer, body composition, along with assessments of heart and lung function, blood pressure, grip strength, a treadmill walking test, and a retinal eye test. Saliva, urine, and 60 mL blood samples are also collected and analyzed for a total of 66 biomarkers at HMC [18].

**Figure 1:**
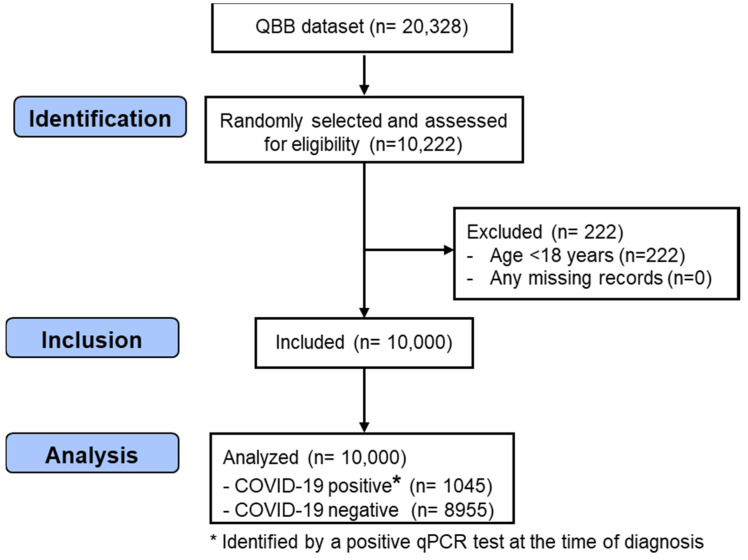
STROBE flow chart illustrating the identification of the included QBB participants. QBB: Qatar Biobank; qPCR: quantitative Polymerase Chain Reaction.

In this study, 10,000 men and women, between the ages of 18 and 79 years, were randomly selected from 20,328 participants recruited by the QBB study. The inclusion criteria were participants > 18 years old and the availability of complete information on the exposure and outcome variables. Participants < 18 years of age (*n* = 222) and participants with missing data on COVID-19 and the exposure variables (*n* = 0) were excluded. Those with complete information on the exposure and outcome variables were eligible for inclusion in this study. Figure 1 shows the process of the selection of the participants from the QBB dataset.

### 2.2. Outcome Variable: COVID-19 Infection

The status of COVID-19 infection was ascertained through laboratory diagnosis (positive qPCR test) at the time of infection [19].

This diagnosis was performed by QBB’s clinical staff, and the COVID-19-positive QBB participants were included in the Qatar COVID-19 biorepository—a national initiative designed to facilitate extensive biomedical research to address the demand for high-quality clinical data on COVID-19 in Qatar. Patients diagnosed with COVID-19 were enrolled during their illness period from three main public hospitals in Qatar. This recruitment took place over a span of 7 months, from March to September 2020. QBB and the Qatar COVID-19 biorepository are linked. In fact, the QBB registry contains two types of COVID-19-positive participants: (1) participants already recruited to the QBB study, and (2) participants who are diagnosed with COVID-19 and have not already ben recruited to the QBB study. The latter group was recruited to the QBB study at the time of positive COVID-19 diagnosis and underwent the same interviews and medical assessments as the existing QBB participants.

### 2.3. Exposure Variables: Sociodemographic and Lifestyle Factors

Various sociodemographic and lifestyle factors were assessed, including age, gender, education level (lower: up to secondary school, intermediate: technical or professional school, but below university level, and higher: university graduate or higher), smoking status (non-smokers, ex-smokers, and current smokers), and physical activity level (metabolic equivalent of task [MET] hours/week, recoded as tertiles), which were collected using questionnaires filled through face-to-face interviews conducted at the QBB clinic by professional nurses. Data were also obtained on history of diabetes and hypertension. Diabetes was defined as a self-reported doctor diagnosis, HbA1c ≥ 6.5%, Random Blood Glucose (RBG) ≥ 11.1 mmol/L, or Fasting Blood Glucose (FBG) ≥ 7 mmol/L. Hypertension was defined as a self-reported doctor diagnosis, Systolic Blood Pressure ≥ 140 mmHg, or Diastolic Blood Pressure ≥ 90 mmHg. Body Mass Index (BMI) was assessed and categorized as normal (18.5–24.9 kg/m^2^), overweight (25–29.5 kg/m^2^), and obese (≥30 kg/m^2^). The dietary habits of the QBB participants were evaluated through a self-administered computer-based food frequency questionnaire (FFQ), comprising 102 food items to assess the frequency of their consumption of various foods and beverages over the past year [18]. Although derived from the European Prospective Investigation into Cancer and Nutrition (EPIC) study, this particular FFQ has not undergone validation among the Qatari population. Nonetheless, the food items in this questionnaire closely align with those of a recently validated FFQ used in Qatar [20]. A history of bariatric surgery and dietary supplement use (multivitamin/minerals, iron, calcium, folic acid, vitamin D, vitamin C, vitamin B, and others) were assessed via a nurse-administered interview, while serum vitamin D levels (ng/mL) were measured.

### 2.4. Statistical Analysis

Quantitative variables were described as mean ± standard deviation (SD) and qualitative (categorical) variables were described as frequencies (counts) and percentages. A student *t*-test and Chi-square test were used to assess the differences in the continuous variables and categorical variables in terms of COVID-19 infection status, respectively. Dietary patterns were identified using a factor analysis. The number of dietary factors to retain was determined by Eigen values > 1, a scree plot, and the interpretability of the identified patterns. Logistic regression was used to examine the association between the exposure variables and COVID-19 infection. Two models were used for the primary analysis and computation of the OR with a 95% confidence interval (CI). Model 1 was adjusted for age and gender, and model 2 was further adjusted for education, smoking, physical activity, BMI categories, diabetes, hypertension, and any supplement use. Associations between COVID-19 and the consumption of various dietary supplements were also assessed by using logistic regression across three multivariable models. Model 3 was similar to model 2, with the exception of an adjustment made for bariatric surgery, as opposed to BMI in Model 2. Subgroup analyses were conducted, and multiplicative interactions between gender, BMI levels, hypertension, diabetes, smoking, and serum vitamin D levels combined with vitamin D supplement use were tested. All the analyses were conducted using STATA software (Version 17, Stata Corporation, College Station, TX, USA) [21], and *p*-values < 0.05 (2-tailed) were considered to be statistically significant.

### 2.5. Ethical Approval

The Institutional Review Boards at QBB (Approval number: EX-2021-QF-QBB-RES-ACC-00065-0181) and Qatar University (QU-IRB 1779-E/22) ethically approved the study. The study sample was obtained from QBB in accordance with the principles outlined in the Declaration of Helsinki, which is designed to safeguard the well-being of human subjects involved in Biomedical Research [22]. QBB obtains informed consent from the participants enrolled in the study, while maintaining anonymity and the non-disclosure of personal identity.

## 3. Results

### 3.1. Sample Characteristics

The mean age of the participants in this study was 40.3 ± 13.1 years, and 52.2% were females. The overall prevalence of obesity and history of bariatric surgery was 44.2% and 12.1%, respectively. Of the included 10,000 participants, 1045 (10.5%) had COVID-19 (Table 1). A descriptive analysis showed that individuals with COVID-19 infection were more likely to be obese, non-smokers, more physically active, and have higher scores for modern dietary pattern. They were also more likely to have a history of bariatric surgery (14.9%) and have reported a higher use of dietary supplements (61.1%) than those without COVID-19. Among vitamin and mineral supplements, the most reported supplements were multivitamin/minerals (37.4%) and vitamin D (22.6%). Supplementary Figure S1 shows the distribution of serum vitamin D in the study population, where the mean serum vitamin D level was 19.29 (ng/mL). Totals of 16.4% and 20.3% of the participants had hypertension and diabetes, respectively (Table 1).

Three major dietary patterns were identified, and their factor loadings are shown in Supplementary Table S1. Accordingly, the foods identified in each pattern were named as modern, prudent, and convenience patterns, respectively. The three dietary patterns explained 33.5% of the variation in food intake, which is comparable to other published studies on dietary patterns in the region [23,24,25]. The modern pattern was characterized by foods high in saturated fat and refined carbohydrates such as fast food, lasagna, mixed dishes of chicken, meat and fish, soft drinks, biryani, desserts, ice cream, chocolate, Asian noodles, zaatar fatayer (thyme and sesame spread on flour dough), croissants, and potatoes. The prudent pattern had high factor loadings of cooked and raw vegetables, fruits, fish, soups and starters, fruit juice, and nuts. The convenience pattern consisted of foods that are ready-to-eat or require minimal preparation, such as yoghurt, cheese, cereal, milkshakes, butter, croissants, zaatar fatayer, and many types of breads (white bread, brown bread, and Arabic bread).

### 3.2. Sociodemographic and Lifestyle Factors and COVID-19 Infection

Among the sociodemographic and lifestyle factors, a statistically significant association of COVID-19 was found with vitamin D, smoking status, obesity, and the modern dietary pattern (Table 2). Vitamin D supplement usage was associated with an 18% reduced likelihood of COVID-19 (OR = 0.82; 95% CI: 0.69–0.97; *p* = 0.022) in the fully adjusted model. Compared to non-smokers, current and ex-smokers had 45% and 30% lower odds of having COVID-19, respectively (*p* < 0.001). Using the normal BMI category as a reference group, participants who were obese were 20% more likely to have COVID-19 infection after adjusting for covariates (OR = 1.20; 95% CI: 1.00–1.45; *p*-value = 0.049). Among the three dietary patterns, the modern dietary pattern was positively associated with COVID-19, and the odds increased by 9% per 1 SD increment in modern dietary pattern score (*p* = 0.012). A history of bariatric surgery was also associated with 24% higher odds of COVID-19 infection (OR = 1.24; 95% CI: 1.03–1.50). No associations were found between gender, education, physical activity, diabetes, hypertension, other dietary supplements, and other dietary patterns with COVID-19 (Table 2 and Table 3).

### 3.3. Vitamin D and COVID-19 Infection

Subgroup analyses revealed no significant interactions between vitamin D supplement use and sociodemographic and lifestyle factors in relation to COVID-19. However, an association between vitamin D supplementation and COVID-19 infection was only observed among those with high serum vitamin D levels (OR = 0.71; 95% CI: 0.53–0.95) or those who did not have hypertension (OR = 0.83; 95% CI: 0.70–1.00) (Supplementary Table S2).

Supplementary Figure S1 presents the distribution of serum levels among the participants. The association between quartiles of serum vitamin D and COVID-19 infection is presented in Table 4. In the fully adjusted model, the ORs (95% CI) for COVID-19 infection, from the lowest to highest quartiles, were 1.00 (reference group), 1.19 (1.00–1.43), 1.06 (0.87–1.29), and 0.98 (0.80–1.20) (*p* = 0.552). We could not detect a significant trend of association between COVID-19 infection and vitamin D serum level quartiles, ranging from high to low serum vitamin D levels.

## 4. Discussion

In this large retrospective cross-sectional analysis of data from 10,000 QBB subjects, we report on the associations between various sociodemographic and lifestyle factors and COVID-19 infection. Dietary patterns were constructed using a factor analysis—a commonly used method in epidemiological research [26]. Smoking (current and past) and vitamin D supplement use were inversely associated with COVID-19 infection, while subjects with obesity, a history of bariatric surgery, and higher modern dietary pattern scores—characterized by foods high in saturated fat and refined carbohydrates (e.g., fast food, soft drinks, and dessert)—had higher odds of COVID-19. No significant associations were found for gender, education, physical activity, diabetes, hypertension, and other dietary supplements or patterns.

### 4.1. Smoking Status and COVID-19 Susceptibility

Lower odds of COVID-19 in the current and former smokers in this sample was an unexpected, albeit not entirely inconsistent, finding. Previous studies have also reported lower rates of smokers among patients diagnosed with COVID-19, compared to non-smokers and former smokers [27,28,29]. The literature has explored the association between smoking and nicotine with SARS-CoV-2 infection. While an upregulation of angiotensin-converting enzyme 2 (ACE2) receptors, which are used by the virus for cell entry, is linked to nicotine exposure [30,31], it is also possible that nicotine binds ACE2 receptors, thus competing with SARS-CoV-2 binding and reducing the virus competence. In fact, several studies have indicated potential preventive effects of nicotine and/or caffeine in COVID-19 by blocking ACE2 receptor binding and preventing SARS-CoV-2 infection [32,33]. Furthermore, several reports have demonstrated the binding of DNA fragments of SARS-CoV-2 Spike protein to nicotinic acetylcholine receptors (nAChRs) [34,35]. This observation supports the notion that COVID-19 can dysregulate the nicotinic cholinergic system, and this dysregulation may be involved in the pathophysiology of COVID-19. In this regard, nicotine and other nicotinic cholinergic agonists may protect nAChRs and thus could have a beneficial role in COVID-19 patients [34,35]. Additionally, nicotine has been reported to attenuate the cytokine storm associated with COVID-19 symptoms [34,36,37]. These observations may potentially explain the incidence of COVID-19 among non-smokers compared to smokers in this study. Alternatively, selection bias could play a role, as symptomatic patients, including smokers with coughs, might undergo more frequent testing, potentially skewing the results. However, the opposite—i.e., increased odds of COVID-19 among smokers—has also been reported in the literature, where smoking has been associated with worse outcomes [38,39]. The contribution of smoking to COVID-19 diagnosis may, in fact, depend on the stage of COVID-19 infection, where smoking may have a protective role at the early stages of infection and a pathogenic role at later stages. These notions remain to be investigated in future studies.

### 4.2. Vitamin D Supplementation and COVID-19 Susceptibility

In this study, COVID-19 was associated with several dietary and nutritional parameters among the Qatari population. Vitamin D supplementation was inversely associated with COVID-19 infection. This is in agreement with many studies that have indicated a protective role of vitamin D against COVID-19 acquisition. For example, vitamin D deficiency has been linked to an increased susceptibility to COVID-19 infection and a higher risk of severe outcomes [40]. A retrospective cohort study found that patients supplemented with vitamin D were at a lower risk of SARS-CoV-2 infection and COVID-19-related mortality [41]. Similarly, a double-blind, parallel randomized controlled trial (RCT), reported that vitamin D supplementation (leading to increased vitamin D serum levels) seemed to protect against SARS-CoV-2 infection, regardless of baseline vitamin D status [42]. In contrast, another RCT reported that individuals with vitamin D insufficiency at baseline who received vitamin D supplementation were not at a lower risk of acute respiratory infections or COVID-19 [43]. In the present study, subgroup analyses showed that vitamin D supplementation was associated with lower odds of COVID-19 in participants with higher serum vitamin D levels. However, no significant interactions between vitamin D supplement use and sociodemographic and lifestyle factors were identified in relation to a positive COVID-19 diagnosis. Vitamin D metabolites play a role in boosting immune responses against respiratory viruses and bacteria [44]. The protective effect of vitamin D against COVID-19 may be explained by the activation of the production of antimicrobial peptides, such as cathelicidin LL-37 and human beta-defensin 2, that can bind to SARS-CoV-2 spike protein, inhibiting its binding to host ACE2 receptors and hindering virus infection [45,46,47].

### 4.3. Obesity and History of Bariatric Surgery and COVID-19 Susceptibility

Obesity was expectedly associated with a higher susceptibility to COVID-19 in this study. Obesity is an independent risk factor for COVID-19 and the existing literature has consistently illustrated that individuals with a BMI of ≥30 kg/m^2^ are more likely to test positive for COVID-19, and experience worse outcomes and higher mortality rates [46]. This is potentially due to obesity-related chronic inflammation, which impairs the immunological responses of the body and its ability to respond to infections [48]. Participants with a history of bariatric surgery had higher odds of contracting COVID-19, although it is worth noting that the observed predisposition to COVID-19 in this study is inconsistent with prior research, where bariatric surgery appeared to be associated with lower mortality rates and a reduced risk of poor outcomes [49,50,51]. The positive association in this study may be partially explained by the fact that bariatric surgery may result in vitamin and mineral deficiencies and malnutrition, which are known risk factors for various health complications [52,53]. Bariatric surgery is also associated with hypercatabolic and immunosuppressive states in individuals, which may contribute to poorer clinical outcomes after contracting SARS-CoV-2 [54,55]. Alternatively, the observed association may be due to selection bias. Patients with bariatric surgery may have regular follow-ups and are therefore more likely to be routinely tested for COVID-19 compared to surgery-free participants.

### 4.4. Modern Dietary Pattern

Similar to obesity and bariatric surgery, an unhealthy eating pattern was also associated with higher odds of COVID-19 in this study. A cross-sectional analysis of the impact of dietary patterns on COVID-19 severity in Iran found a positive association of an unhealthy diet with longer hospital stays and worse symptoms in COVID-19 patients [56]. Existing research on the associations of COVID-19 and dietary patterns has primarily reported on plant-based diets, which are associated with a lower COVID-19 infection risk [57] and severity [58,59]. Studies have reported that improving the quality of nutritional intake may reduce the impact of infectious diseases [60,61], partly through arachidonic or linoleic acid that suppress viral replication [62]. Additionally, some B vitamins (e.g., vitamin B6, B12, and folate), vitamins C, D, E, and A, omega-3 fatty acids and amino acids, and the polyphenols found in plant-based foods play crucial roles in the functioning of the immune system and the release of inflammatory mediators [11,62,63]. Unhealthy eating habits, such as the modern eating pattern described in this study, lack these potentially protective food elements, which may explain the observed association between modern eating habits and increased rates of COVID-19. Moreover, consuming high-fat diets triggers the activation of the body’s natural immune response and hinders the adaptive immune system, resulting in a chronically inflamed state and compromised protection against viral infections [64]. Personal attitudes and beliefs seem to favor the use of vitamin D and plant-based medicinal products in the treatment or prevention of COVID-19 [65,66], underscoring the role of traditional foodstuffs and dietary recipes in boosting health and curbing infections.

### 4.5. Strengths and Limitations

To the best of our knowledge, this is the first study to assess the association between COVID-19 and lifestyle and sociodemographic factors in Qatar. This study has some notable strengths. Firstly, the large sample size increases the precision of estimates. Secondly, participants were selected randomly. Owing to the detailed collection process of QBB data, which was started before the COVID-19 pandemic, we were able to adjust for numerous confounding factors; reverse causation is unlikely in the study. Finally, the 102-item FFQ allowed for capturing the most commonly consumed foods in the local population.

However, due to the observational nature of this study and the secondary use of data, no temporality or causality could be established. Additionally, the FFQ questionnaire that was employed was qualitative and did not include portion sizes. This method of diet recall is associated with some measurement errors, notably recall bias.

## 5. Conclusions

In conclusion, COVID-19 in this Qatari population was inversely associated with smoking (current or past) and vitamin D supplementation. On the contrary, subjects with obesity, a history of bariatric surgery, and higher scores for the modern dietary pattern were more likely to have a positive COVID-19 diagnosis. Overall, this study contributes to the growing body of knowledge on COVID-19 infection and sheds light on some of the factors possibly associated with increased rates of COVID-19, and potentially other respiratory infections. While the associations of lifestyle and sociodemographic factors with COVID-19 are fairly supported by sound evidence, differences in COVID-19 infection rates remain difficult to determine and prove, warranting future investigation. Further research, particularly long-term prospective cohort studies, are needed to assess the temporal relationships between potential risk or protective factors and a COVID-19 diagnosis. Additionally, the high rates of obesity and bariatric surgery in this population, coupled with their connection to COVID-19 infection, highlight the importance of implementing effective public health measures to address the growing obesity epidemic. 

## Figures and Tables

**Table 1:** Sample characteristics by COVID-19 infection status.

	Total	No	Yes	*p*-Value
	*N* = 10,000	*N* = 8955	*N* = 1045	
Age (years)	40.3 ± 13.1	40.4 ± 13.1	39.6 ± 12.7	0.083
Gender				<0.001
Male	4780 (47.8%)	4351 (48.6%)	429 (41.1%)	
Female	5220 (52.2%)	4604 (51.4%)	616 (58.9%)	
Educational level				0.020
Low	1796 (18.0%)	1594 (17.8%)	202 (19.3%)	
Medium	3040 (30.4%)	2694 (30.1%)	346 (33.1%)	
High	5164 (51.6%)	4667 (52.1%)	497 (47.6%)	
Smoking status				<0.001
None	6468 (64.7%)	5695 (63.6%)	773 (74.0%)	
Smoker	1866 (18.7%)	1735 (19.4%)	131 (12.5%)	
Ex-smoker	1666 (16.7%)	1525 (17.0%)	141 (13.5%)	
Leisure time physical activity (MET hours/week)	24.9 ± 52.5	24.9 ± 51.0	24.3 ± 64.2	0.74
Modern dietary pattern score	0.0 ± 1.00	−0.01 ± 1.0	0.1 ± 1.1	0.001
Prudent dietary pattern score	0.0 ± 1.00	−0.0 ± 1.0	0.02 ± 0.9	0.45
Convenience dietary pattern score	0.0 ± 1.00	−0.01 ± 1.0	0.1 ± 1.1	0.032
Serum vitamin D (ng/mL)	19.3 ± 11.1	19.3 ± 1.1	18.9 ± 10.9	0.22
Hypertension	1638 (16.4%)	1479 (16.5%)	159 (15.2%)	0.28
Diabetes	2026 (20.3%)	1808 (20.3%)	218 (21.0%)	0.60
History of bariatric surgery	1213 (12.1%)	1058 (11.8%)	155 (14.9%)	0.004
BMI categories				0.051
Normal	2112 (21.1%)	1905 (21.3%)	207 (19.8%)	
Overweight	3462 (34.7%)	3123 (34.9%)	339 (32.4%)	
Obese	4417 (44.2%)	3918 (43.8%)	499 (47.8%)	
Dietary supplement use				
Multivitamin/minerals	3565 (35.6%)	3174 (35.4%)	391 (37.4%)	0.21
Calcium	726 (7.3%)	649 (7.2%)	77 (7.4%)	0.89
Folic acid	316 (3.2%)	276 (3.1%)	40 (3.8%)	0.19
Iron	1366 (13.7%)	1208 (13.5%)	158 (15.1%)	0.15
Vitamin B	806 (8.1%)	728 (8.1%)	78 (7.5%)	0.45
Vitamin C	665 (6.7%)	604 (6.7%)	61 (5.8%)	0.27
Vitamin D	2402 (24.0%)	2166 (24.2%)	236 (22.6%)	0.25
Other supplements	3315 (33.1%)	2968 (33.1%)	347 (33.2%)	0.97
Any supplement use	5977 (59.8%)	5339 (59.6%)	638 (61.1%)	0.37

Data are presented as mean ± SD for continuous measures, and *n* (%) for categorical measures. For the dietary patterns, the numbers represent mean dietary pattern scores ± SD with a mean factor score as zero, and an SD of 1 after factor analysis (Supplementary Table S1). Higher dietary mean scores are present in participants with COVID-19, and lower scores in those without COVID-19. MET: metabolic equivalent of task.

**Table 2:** Association between sociodemographic and lifestyle factors with COVID-19 infection (*n* = 10,000).

	Model 1		Model 2	
	OR [95% CI]	*p*-Value	OR [95% CI]	*p*-Value
Vitamin D	0.81 (0.68–0.96)	0.018	0.82 (0.69–0.97)	0.022
Age (years)	0.99 (0.99–1.00)	0.050	0.99 (0.99–1.00)	0.084
Gender				
Male	1.00		1.00	
Female	1.03 (0.88–1.21)	0.715	1.03 (0.88–1.21)	0.704
Education				
Low	1.00		1.00	
Medium	0.95 (0.77–1.17)	0.641	0.94 (0.76–1.17)	0.588
High	0.86 (0.71–1.04)	0.124	0.85 (0.70–1.03)	0.096
Smoking				
Non	1.00		1.00	
Smoker	0.55 (0.44–0.68)	<0.001	0.55 (0.44–0.68)	<0.001
Ex-smoker	0.70 (0.57–0.86)	<0.001	0.70 (0.57–0.86)	<0.001
Leisure time PA (MET hours/week)				
T1	1.00		1.00	
T2	1.02 (0.87–1.20)	0.817	1.03 (0.88–1.21)	0.737
T3	0.94 (0.79–1.11)	0.472	0.93 (0.79–1.10)	0.404
BMI level				
Normal	1.00			
Overweight	1.09 (0.90–1.32)	0.379		
Obese	1.20 (1.00–1.45)	0.049		
Diabetes				
No	1.00		1.00	
Yes	1.09 (0.91–1.31)	0.338	1.10 (0.92–1.32)	0.308
Hypertension				
No	1.00		1.00	
Yes	0.94 (0.76–1.16)	0.547	0.96 (0.78–1.18)	0.693
Modern dietary pattern	1.09 (1.02–1.16)	0.012	1.08 (1.01–1.16)	0.018
Prudent dietary pattern	1.03 (0.97–1.10)	0.344	1.03 (0.97–1.10)	0.307
Convenience dietary pattern	1.05 (0.99–1.12)	0.100	1.05 (0.99–1.12)	0.092
Any supplement use	1.12 (0.96–1.30)	0.155	1.10 (0.95–1.28)	0.205
History of bariatric surgery				
No			1.00	
Yes			1.24 (1.03–1.50)	0.022

All the variables in the model were mutually adjusted. BMI: body mass index; CI: confidence interval; MET: metabolic equivalent of task; OR: odds ratio; PA: physical activity; and T: tertile.

**Table 3:** Association between dietary supplement use and COVID-19 infection (*n* = 10,000).

	Model 1		Model 2		Model 3	
	OR (95% CI)	*p*-Value	OR (95% CI)	*p*-Value	OR (95% CI)	*p*-Value
Any supplement use	1.00 (0.87–1.14)	0.979	1.03 (0.90–1.19)	0.652	1.02 (0.89–1.18)	0.749
Multivitamin/minerals	1.07 (0.94–1.22)	0.322	1.17 (0.99–1.40)	0.067	1.16 (0.97–1.38)	0.096
Calcium	0.96 (0.75–1.22)	0.723	0.97 (0.75–1.25)	0.792	0.96 (0.75–1.24)	0.775
Folic acid	1.13 (0.80–1.59)	0.485	1.18 (0.83–1.66)	0.356	1.17 (0.82–1.65)	0.385
Iron	1.01 (0.84–1.22)	0.886	1.02 (0.84–1.24)	0.869	1.00 (0.82–1.22)	1.000
Other supplements	0.94 (0.81–1.08)	0.357	0.90 (0.76–1.06)	0.197	0.90 (0.76–1.06)	0.216
Vitamin B	0.87 (0.68–1.12)	0.281	0.89 (0.69–1.14)	0.348	0.87 (0.68–1.12)	0.288
Vitamin C	0.84 (0.64–1.10)	0.210	0.86 (0.65–1.14)	0.288	0.86 (0.65–1.13)	0.276
Vitamin D	0.85 (0.73–1.00)	0.046	0.81 (0.68–0.96)	0.018	0.82 (0.69–0.97)	0.022

Model 1 adjusted for age and gender. Model 2 further adjusted for education, smoking, physical activity, BMI, diabetes, hypertension, dietary patterns, and any supplement use. Model 3 was adjusted for bariatric surgery instead of BMI in Model 2. Both Models 2 and 3 adjusted for dietary patterns.

**Table 4:** Odds ratio (95% CI) for COVID-19 infection by quartiles of serum vitamin D.

	Quartiles of Serum Vitamin D				
	Q1	Q2	Q3	Q4	*p*-Value
Model 1	1.00	1.19 (0.99–1.42)	1.04 (0.85–1.26)	0.94 (0.77–1.15)	0.324
Model 2	1.00	1.19 (1.00–1.43)	1.06 (0.87–1.29)	0.98 (0.80–1.20)	0.552

Model 1 adjusted for age and gender. Model 2 further adjusted for education, smoking, physical, BMI, diabetes, hypertension, and any supplement use.

## Data Availability

Data are contained within the article.

## Supplementary Materials

The following supporting information can be downloaded at: https://www.mdpi.com/article/10.3390/nu16071037/s1, Figure S1: Distribution of serum levels of vitamin D.; Table S1: Factor loadings of the identified dietary patterns; Table S2: Subgroup analyses of the association between vitamin D supplement use and COVID-19 infection.
