# Supplementary material for: The Association between Lifestyle Factors and COVID-19: Findings from Qatar Biobank
Source: Nutrients. 2024 Apr 3;16(7):1037. doi: 10.3390/nu16071037 (PMC11013885; doi:10.3390/nu16071037)
Supplement: Supplementary file 1 [file nutrients-16-01037-s001.zip › nutrients-2729594-supplementary.pdf]

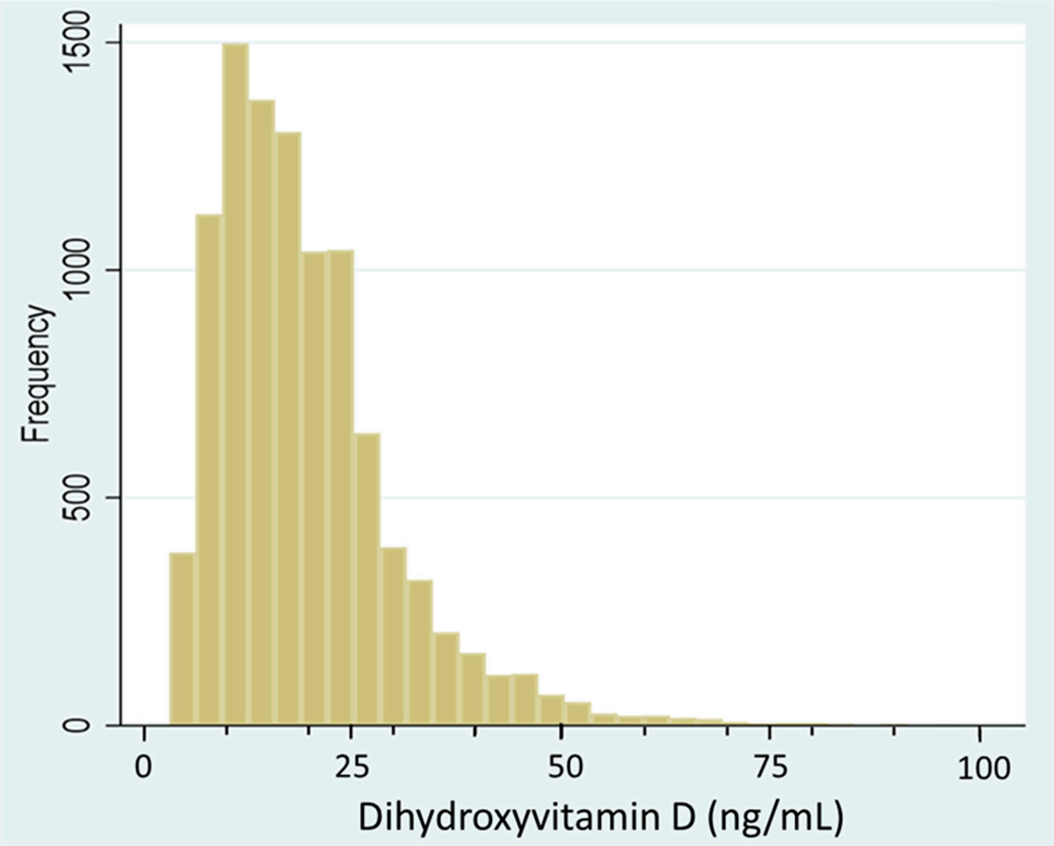

Supplementary Figure S1. Distribution of serum levels of vitamin D.

Supplementary Table S1. Factor loadings of identified dietary patterns.

Variable	Modern pattern	Prudent pattern	Convenience pattern
Fast food	0.73		
Lasagna	0.64		
Chicken/meat fish mixed dish	0.62	0.43	
Soft drink	0.61		
Biryani	0.58	0.31	
Desserts	0.57		0.23
Ice cream	0.54		
Chocolate	0.52		
Asian noodle	0.51	0.27	
Zaatar fatayer	0.47		0.45
Croissant	0.47		0.44
Potato	0.42	0.34	
White rice	0.39	0.31	
Red meat	0.35	0.30	
Chicken	0.35	0.25	
Salad and raw vegetables		0.69	
Fresh fruit		0.69	
Grilled/fried/baked Fish		0.61	
Canned/dried fruit and dates		0.61	

Fish		0.60	
Salad and cooked vegetables	0.23	0.55	0.22
Soups/starters	0.40	0.52	0.30
Fresh fruit juice	0.22	0.51	
Nuts		0.39	
Eggs		0.34	
Yoghurt			0.61
Cheese			0.55
Milk added to cereal			0.53
Milk shakes			0.53
Milk			0.45
Butter			0.44
White bread	0.42		0.43
Other bread	0.30		0.40
Brown bread		0.32	0.37
Arabic/Iranian bread	0.32		0.34
Breakfast Cereal			0.33
Coffee			0.30
Tea		0.21	0.29
Variance explained (%)	22.2	6.1	5.2

Absolute values of factor loadings above 0.20 are displayed in the figure only.

Supplementary Table S2. Subgroup analyses of the association between vitamin D supplement use and COVID-19 infection.

	**Vitamin D supplement use**		**P value**	**P for interaction**
	No	Yes		
Sex				0.789
Male	1.00	0.80 (0.59-1.09)	0.155	
Female	1.00	0.88 (0.73-1.06)	0.169	
BMI levels				0.576
Normal	1.00	0.88 (0.61-1.27)	0.499	
Overweight	1.00	0.91 (0.69-1.20)	0.488	
Obese	1.00	0.80 (0.64-1.01)	0.060	
Quartiles of serum vitamin D				0.117
Q1	1.00	0.58 (0.32-1.04)	0.068	
Q2	1.00	0.94 (0.69-1.28)	0.697	
Q3	1.00	1.07 (0.80-1.43)	0.660	
Q4	1.00	0.71 (0.53-0.95)	0.023	
Diabetes				0.244
No	1.00	0.89 (0.75-1.07)	0.213	
Yes	1.00	0.74 (0.53-1.03)	0.072	

Hypertension				0.663
No	1.00	0.83 (0.70-1.00)	0.044	
Yes	1.00	0.95 (0.66-1.36)	0.770	
Smoking				0.655
None	1.00	0.87 (0.73-1.04)	0.125	
Smoker	1.00	0.69 (0.37-1.28)	0.239	
Ex-smoker	1.00	0.85 (0.52-1.37)	0.502	

Values are odds ratio (95% CI). Models adjusted for age and gender, education, smoking, physical, BMI, diabetes, hypertension, and dietary patterns. Stratification variables were not adjusted in the corresponding models.
